# Distribution and risk assessment of microplastics in a source water reservoir, Central China

**DOI:** 10.1038/s41598-024-84894-z

**Published:** 2025-01-02

**Authors:** Minghui Shen, Yang Li, Liwen Qin, Xudong Chen, Tianyu Ao, Xishu Liang, Kaibo Jin, Yanyan Dou, Juexiu Li, Xuejun Duan

**Affiliations:** School of Smarts Energy and Environment, Zhongyuan University of Technology, Zhengzhou, 450007 China

**Keywords:** Microplastics, Source water reservoir, Risk assessment, Correlation analysis, Environmental sciences, Limnology

## Abstract

The current researches on microplastics in different water layers of reservoirs remains limited. This study aims to investigate the microplastics in different water layers within a source water reservoir. Results revealed that the abundance of microplastics ranged from 2.07 n/L to 14.28 n/L (reservoir, water) and 3 to 7.02 n/L (river, water), while varied from 350 to 714 n/kg_(dw)_ (reservoir, sediment) and 299 to 1360 n/kg_(dw)_ (river, sediment). The average abundance in surface, middle, and bottom water were 6.83 n/L, 6.30 n/L, and 6.91 n/L respectively. Transparent fibrous smaller than < 0.5 mm were identified as the predominant fraction with Polypropylene and Polyethylene being the prevalent polymer types. Additionally, the pollution load index, hazard index, and pollution risk index were calculated for different layers and sediments. Results showed that surface water exhibited a moderate level of risk while the sediments posed a low level of risk. Both the middle and bottom water showed elevated levels of risk due to higher concentrations of polymers with significant toxicity indices. This study presents novel findings on the distribution of microplastics in different water layers, providing crucial data support for understanding the migration patterns of microplastics in source water reservoirs and facilitating pollution prevention efforts.

**Supplementary Information:**

The online version contains supplementary material available at 10.1038/s41598-024-84894-z.

## Introduction

The affordability, high strength, lightweight nature, and strong durability of plastic render it extensively utilized in various domains of daily life as well as industrial and agricultural production. However, the global issue of environmental pollution caused by plastic waste has arisen due to both the imperfect management system for handling plastic waste and its inherent difficulty in degradation^1^. According to certain researchers’ projections, the quantity of plastic waste entering the environment due to inadequate management is anticipated to increase by approximately 600-fold compared to 1950 by the year 2060, and it is estimated that by 2060, an annual influx of 265 million tons of plastic waste will be introduced into the environment^2,3^. The exposure of plastic waste to sunlight and weathering in the natural environment can result in the formation of microplastics (MPs) measuring less than 5 mm, while studies have demonstrated its inherent ecological harm, as well as its potential role as a carrier for dissolved pollutants, thereby exacerbating environmental pollution levels^4,5^. Early investigations on MPs primarily focused on marine ecosystems^6^, while in recent years, there has been a growing interest in the contamination of MPs in freshwater environments. However, the current research on the pollution of MPs in water sources remains limited, presenting challenges for establishing comprehensive environmental standards in this context, which continues to be a focal point of our investigation^7^.

For the past years, reservoirs have emerged as a primary source of water supply for numerous urban and rural areas. Reservoir is typically created by an artificial dam, rendering it a lake in a “semi-artificial and semi-natural state”. The presence of a deeper water column and longer hydraulic retention times often lead to the occurrence of aggregation phenomenon, thereby establishing reservoirs as crucial “sinks” for MPs^8,9^. Previous studies have predominantly focused on investigating organic matter, such as nitrogen and phosphorus, along with heavy metals like iron and manganese in reservoirs^10,11^, while MPs, as an emerging contaminant, our current knowledge regarding the pollution characteristics and mechanisms in source water reservoirs remains limited. There have been reports of the occurrence of MPs detected in certain reservoirs within China, including Danjiangkou Reservoir^12^, Chitian Reservoir^13^, Three Gorges Reservoir^14^, and Liujiaxia Reservoir^15^. Results revealed that the abundance of MPs in these reservoirs ranged from 0.53 to 24.8 n/L, and a comprehensive analysis was conducted on the color, size, and polymer composition of MPs present in the reservoirs and the highest component was found to be transparent fibrous MPs smaller than 1 mm in size. Study conducted by Lin et al. found that the average abundance of MPs in the middle layer water of Danjiangkou Reservoir was significantly higher than that in surface and bottom layer water and there was a change in the color of MPs from the surface to the bottom water layer^12^. The future development of effective policies for the prevention and management of MPs pollution relies on a comprehensive understanding of the levels and migration characteristics of MPs in source water reservoirs.

The safety of drinking water is intricately intertwined with people’s daily lives, and as the primary source of potable water, the quality requirements for drinking water sources are more stringent. After analyzing over 150 samples collected from five continents, researchers discovered that the proportion of MPs in raw water for drinking water treatment plants exceeded 80%^16^. And MPs also have been found in heavily protected drinking water sources in inland southern China which has raised additional concerns regarding its potential impact on public health^17^. However, the current dearth of evaluation data on MPs risk in water sources, particularly in source water reservoirs, remains a significant limitation. On the other hand, the exceptional hydrophobicity, extensive surface area, and enduring nature of plastics facilitate the accumulation of elevated concentrations of organic pollutants on the outermost layer of MPs^18,19^, which can result in a synergistic amplification of pollution, exacerbating its effects. In order to further grasp the extent of MPs pollution, researchers use pollution load index (PLI), pollution risk index (PRI) and so on to assess the ecological risk for freshwater^20–22^. However, the current lack of effective unified standards for the analysis, detection, and risk assessment of MPs remains a significant challenge. Therefore, it is imperative to report additional fundamental research data to provide essential support for the establishment of comprehensive and standardized protocols.

In this study, the Yanhekou Reservoir, situated in the Yangtze River Basin, was selected as the research subject to investigate the spatial distribution of MPs in both water and sediment. Field sampling was conducted to examine the spatial variation in MPs distribution across different water depths. MPs were observed, counted, and detected for their abundance, shapes, sizes, colors, and polymer types in both water and sediment samples. Furthermore, the present study also conducted an ecological risk assessment on MPs found in the reservoir. These findings provide valuable fundamental research data regarding the potential harm caused by MPs.

## Materials and methods

###  Study area and field sampling

**Fig. 1:**
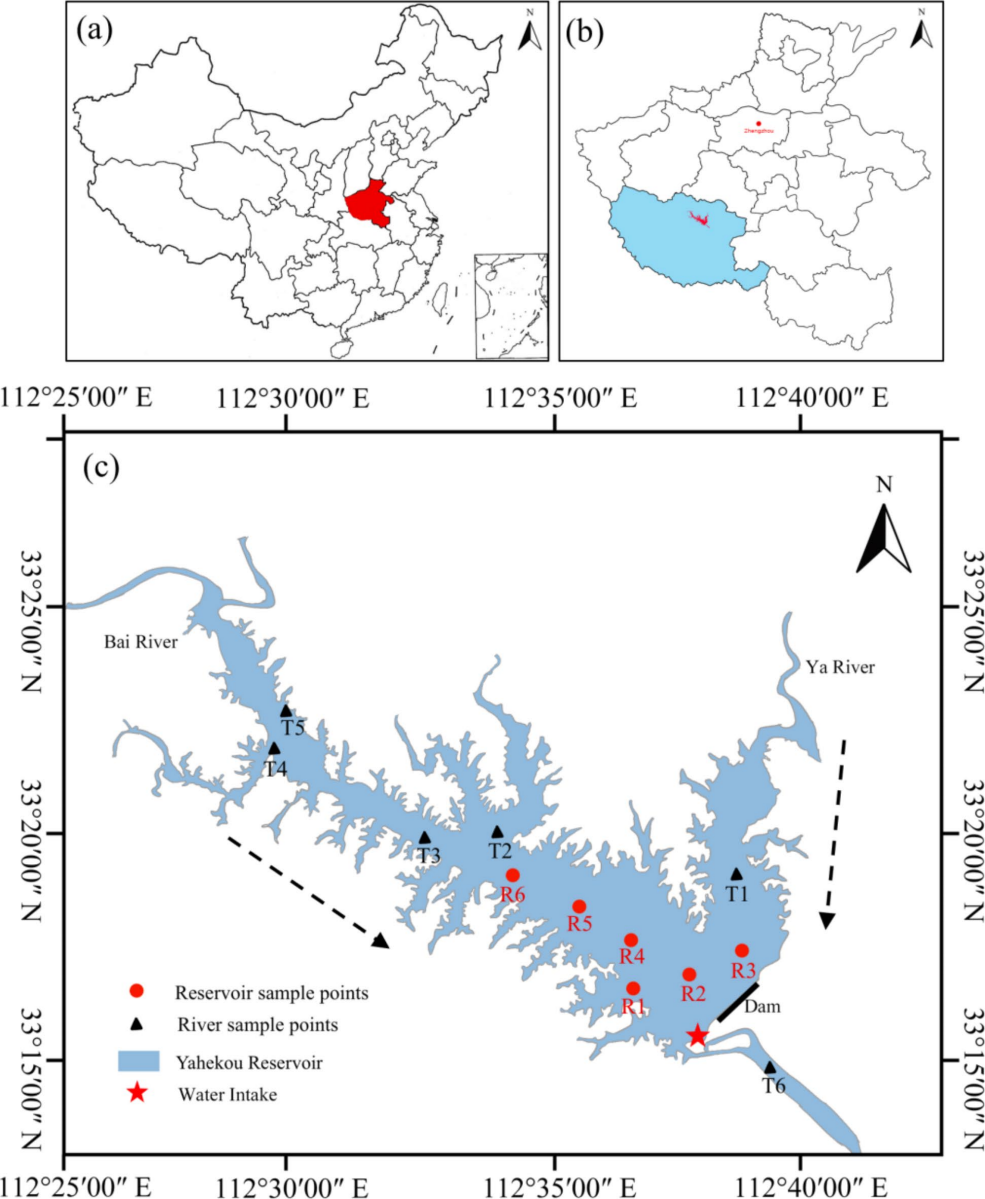
Location of sampling points in study area (This figure was drawn by Procreate software, version 5.2.6, URL: https://procreate.com/cn).

Yahekou Reservoir (Fig. 1) is a large canyon-shaped reservoir located in the upper reaches of Baihe River, a secondary tributary of the Yangtze River, approximately 40 km north of Nanyang City in Henan Province, middle China. This reservoir is a large-scale water management project that integrates flood control, hydropower generation, water supply, and agricultural irrigation which serves as a crucial raw water source for Nanyang City^23^. When the reservoir is at full capacity, it exhibits a surface area of 120 km^2^ and a total volume of 13.39 ×  10^8^ m^3^, with mean and maximum depths measuring 15 and 32 m respectively. The Bai River, originating from the Funiu Mountains, serves as the primary tributary to the reservoir, while another tributary, namely the Ya River, converges with the main reservoir in close proximity to the dam.

In this study, 6 sample points were set in the main reservoir area (R1 to R6), and another 6 sample points were set at different river locations (T1 to T6). The sampling points were accurately determined using a handheld GPS instrument. The latitude and longitude information is presented in Table S1. Surface, middle, and bottom water samples were collected for R1 to R6, while only surface water samples were collected for T1 to T6. The depths of bottom water at points R1 to R6 are 11 m, 16 m, 15 m, 20 m, 15 m, and 10 m, respectively. The depth of middle water is equivalent to half the depth of the bottom water. Sediment samples were collected at all sampling points except for the T3. The collection of samples was completed in August 2023. To collect water samples, a hand-held peristaltic pump with a silicone hose was utilized to obtain 5 L of water at various depths. On-site, the collected samples were filtered through a stainless steel screen with a diameter of 0.054 mm, and the retained material on the screen was thoroughly rinsed with ultra-pure water into a sealed 500 ml brown glass bottle for preservation. The collection of sediment samples involved the retrieval of approximately 1 kg of surface sediment using a Petersen-type sediment grab bucket, which was subsequently placed into an aluminum foil bag for sealing and storage. All collected samples were subsequently transported to the laboratory for further experimentation.

###  Sample pretreatment

The pretreatment methods recommended by the National Oceanic and Atmospheric Administration (NOAA)^24^. All water samples were filtered again through a stainless steel screen with a diameter of 0.054 mm in laboratory and the retained material was thoroughly rinsed with absolute ethyl alcohol into a 200 ml glass beaker respectively. These beakers were subsequently dried in an oven set at 50 ℃, and then 30% hydrogen peroxide were added and placed in a water bath at 50 ℃, where they were left for 48 h for digestion. The solution was ultimately subjected to filtration using a vacuum filter and a 0.45 μm glass fiber filter membrane, with the resulting membrane being carefully preserved in a glass culture dish for subsequent detection.

For sediment samples, large stones or leaves were meticulously removed using stainless steel tweezers and mixed firstly. Subsequently, 100 g of sediment was accurately weighed and transferred into a 1000 ml glass beaker. Following this, 500 ml of saturated NaCl solution was added to the beaker and thoroughly mixed. The mixture was then left undisturbed for duration of 24 h and transferred the supernatant to another beaker. The extraction step was repeated three times consecutively. The supernatant was filtered through a stainless steel screen with a diameter of 0.054 mm and substance trapped on the screen was then rinsed into a glass beaker employing anhydrous ethanol and subjected to drying in an oven at 50 °C. The following steps were the same as those for water samples. In addition, 100 g of sediment were used to measure the moisture content in each sediment sample by the oven drying at 105 °C for 12 h.

###  Detection of MPs

The membranes after filtration were placed on the stage of the microscope (Motic, BA310Digital, China) connected to computer by a camera. Image acquisition software (Motic Images Plus 3.0 ML, China) was used to collect the suspected MPs on the membranes. The amount, size, morphology, and color of the suspected MPs were recorded. The location of suspected MPs was determined by the membrane’s grid. Then the polymer types of the MPs were detected using a Confocal Raman Microscopy Spectrometer (Ideaoptics, gora-Lite, China). The experimental parameters of this spectrometer were set to a wave number range of 60–3500/cm, with a resolution of less than 5/cm and a laser excitation wavelength of 785 nm. For the water samples, the suspected substances on the membranes were detected one by one, and the results were analyzed with the corresponding software (gora. Dawn v1.0). The measured spectrum was considered to be the same as a standard spectrum only when the spectral similarity was greater than 70.0%^25^. After detection, the suspected MPs ultimately identified as MPs were counted for further analyzed.

### Risk assessment method

Unfortunately, a unified risk assessment system for MPs is currently lacking. In consideration of the same Yangtze River basin, we had referred to Xu et al.‘s methods for risk assessment of MPs in the Three Gorges reservoir in this study^14^. The pollution load index (*PLI*) of MPs was determined through the utilization of formulas (1) and (2).

1 $$PLI={{{C_i}} \mathord{\left/ {\vphantom {{{C_i}} {{C_{oi}}}}} \right. \kern-0pt} {{C_{oi}}}}$$

2 $$PL{I_{Zone}}=\sqrt[n]{{PL{I_1} \times PL{I_2} \times \cdots PL{I_n}}}$$

*C*_*i*_ is the abundance of MPs at sample point *i*; *C*_*oi*_ is the minimum abundance of MPs at different water layer and sediment; *n* is the number of sample points; *PLI*_*Zone*_ is the pollution load index of MPs in the calculated zone. The surface, middle, and bottom water bodies as well as sediment were categorized into distinct zones for the purpose of computation in this study. The calculated results were presented in Table S2.

As widely acknowledged, MPs encompass a diverse range of polymer types, each exhibiting varying levels of toxicity in water. Hence, relying solely on the PLI for evaluating the risk posed by MPs in a source water reservoir entails certain limitations. So we incorporated hazard index (H) and pollution risk index (PRI) as additional parameters to enhance the assessment of MPs pollution levels in Yahekou Reservoir^15,26^. The specific calculation is shown in Formula (3)–(6).

3 $${H_i}=\sum {{P_n} \times {S_n}}$$

4 $${H_{Zone}}=\sqrt[n]{{{H_1} \times {H_2} \times \cdots {H_n}}}$$

5 $$PR{I_i}={H_i} \times PL{I_i}$$

6 $$PR{I_{Zone}}=\sqrt[n]{{PR{I_1} \times PR{I_2} \times \cdots PR{I_n}}}$$

*H*_*i*_ is the hazard index of MPs at sample point *i*; *P*_*i*_ is the proportion of different polymer types identified at each sampling point; Si is the toxicity levels of various polymers (Table S3); *H*_*Zone*_ is the hazard index of MPs in the calculated zone; *PRI*_*i*_ is the pollution risk index at sample point *i*; *PRI*_*Zone*_ is the pollution risk index of MPs in the calculated zone. Similarly, relevant calculations were conducted separately for surface, middle, and bottom water bodies as well as sediments.

### Quality control

The glass bottles and aluminum foil bags for storing water samples and sediment samples were thoroughly cleaned with ultra-pure water and dried in oven before the sampling process. At the sampling site, we stored them in a special sealed storage box. In laboratory experiments, the operator donned a cotton lab coat and utilized disposable nit-rile gloves. All laboratory vessels used in the experiment were washed with ultra-pure water and dried by the oven and wrapped tightly in aluminum foil. To prevent contamination from external sources, various items were wrapped in aluminum foil between each step of the experimental operation. Additionally, all laboratory operations such as digestion, extraction, and filtration were conducted within a fume hood. Additionally, three blank experiments were conducted on the ultra-pure water utilized in this study, with each experiment employing 5 L of ultra-pure water. The findings revealed the detection of only 5 MPs in the total 15 L of ultra-pure water, suggesting that the contamination in ultra-pure water may be considered negligible.

### Data analysis

Figure 1 utilized the standard service system (http://bzdt.ch.mnr.gov.cn) as a base in the standard map, employed Procreate software (version 5.2.6) for sampling point information and contour drawing. The data obtained by the spectrometer was subsequently corrected using MATLAB 2020a software. Subsequently, data collection and calculations were performed using office 2010, followed by plot processing with Origin 2018 software. Furthermore, one-way analysis of variance (ANOVA) was conducted employing SPSS Statistics 26 software.

## Results and discussion

### Abundance of MPs

**Fig. 2:**
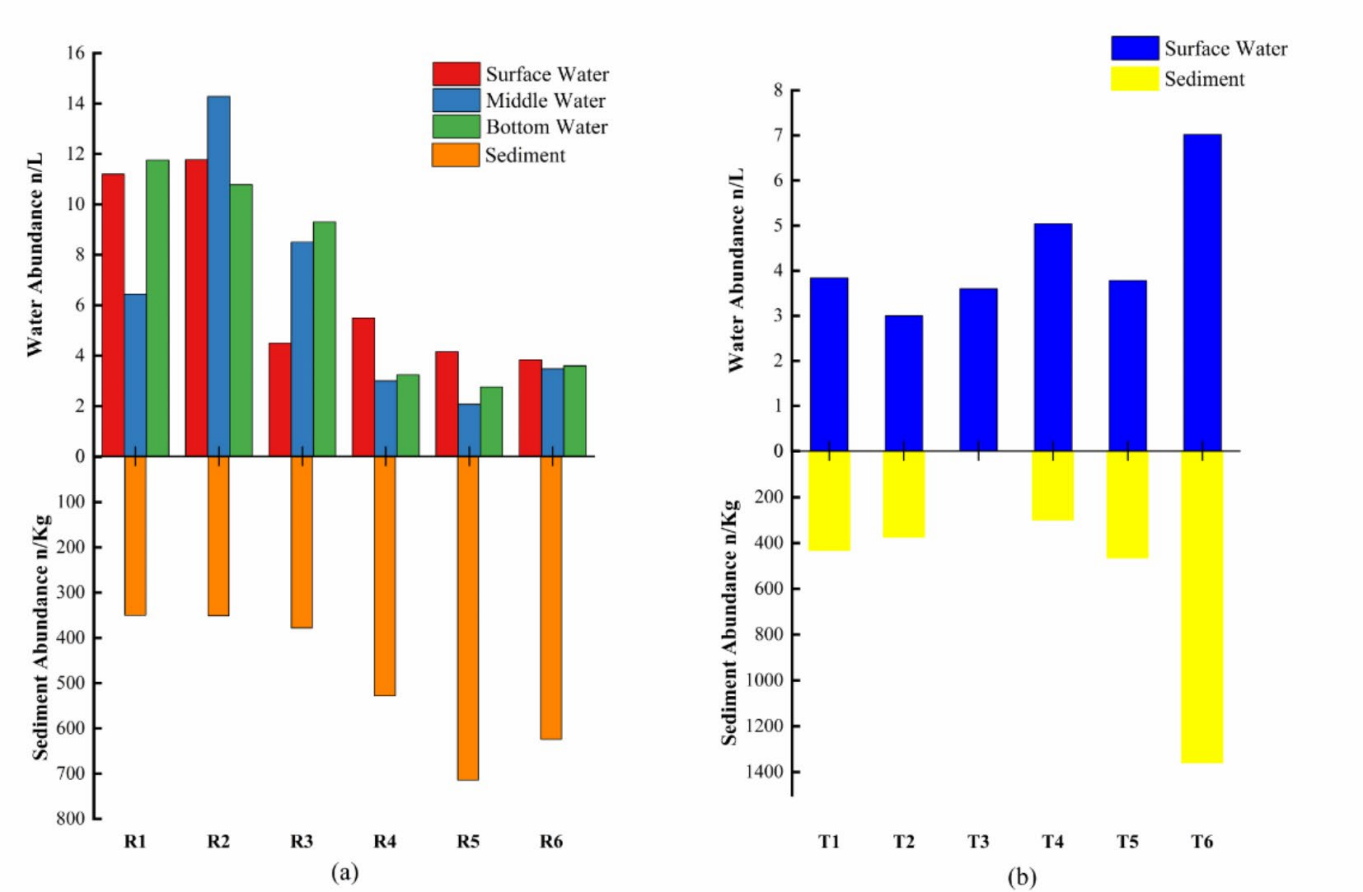
Abundance of MPs in water and sediment samples (**a**: reservoir, **b**: river).

MPs were detected in all water and sediment samples (no sediment was collected at T3). The abundance of MPs in water samples collected from R1 to R6 ranged from 2.07 n/L to 14.28 n/L, with an average abundance value of 6.68 n/L (Fig. 2a). From the perspective of different water layers, the abundance ranges of MPs in surface, middle, and bottom water were 3.84 n/L to 11.77 n/L, 2.07 n/L to 14.28 n/L, and 2.76 n/L to 11.76 n/L, respectively. The average abundances were 6.83 n/L, 6.30 n/L, and 6.91 n/L for different water layers. The abundance of MPs in the bottom water of Yahekou reservoir was observed to be the highest among the three water layers and then was the surface water. The abundance of MPs in the river water samples ranged from 3.00 n/L to 7.02 n/L, with an average abundance of 4.38 n/L. Notably, T6 exhibited the highest abundance at 7.02 n/L. Our field sampling also revealed that T6 is surrounded by densely populated town, suggesting a significant influence of human activities on the presence of MPs.

The overall abundance of MPs in the water at the three sampling points R1, R2, and R3 was higher, particularly in the area where water cruise activities were most intensive within the Yahekou Reservoir. Lin et al.‘s study on Danjiangkou Reservoir also highlighted the significant contribution of aquatic recreational activities to the influx of MPs into the reservoir^12^. Additionally, the three sampling points were situated in close proximity to the dam, resulting in a considerably sluggish water flow rate. Notably, certain studies have demonstrated a significant negative correlation between water flow velocity and abundance of MPs^27^. Liu et al. also highlighted that the dam front represents a potential enrichment zones for MPs accumulation within the reservoir^8^. It was observed that the abundance of MPs in surface, middle, and bottom water at R2 sampling sites exhibited higher levels. This outcome can be attributed to two factors. Firstly, apart from water boating activities, R2 points are also situated along the “midstream of channel” of the upstream inflow, which collectively contribute to this scenario. According to the water intake location, it is evident that the R2 point represents the confluence of the Bai River and Ya River, with a water depth of 16 m and proximity to the dam, thereby creating favorable conditions for MPs accumulation. As upstream water enters the main reservoir, it typically descends into the middle or bottom layers through density current. This phenomenon contributes to the elevated abundance of MPs in the middle water at R2 point. Furthermore, due to these aforementioned factors, both middle and bottom waters exhibit higher abundances compared to surface waters at R3 (Fig. 2a). In the study of Danjiangkou Reservoir, the abundance of MPs was similarly reported to be higher in the mid-water layer than in the surface and bottom layer^12^. The effects of different geographical locations on the abundance of MPs in water bodies were examined using one-way ANOVA. The results revealed a statistically significant difference in abundance of MPs across various locations (F (5, 4.78) = 11.38, *p* < 0.05).

The vertical distribution of MPs in water revealed that the highest average abundance was observed in the bottom water. Slow water flow rate in the main reservoir promoted a longer hydraulic retention time, facilitating the vertical settlement of MPs^8^. However, the release of MPs from sediments into the water constituted another significant factor that should not be disregarded. As we all know, the endogenous pollution caused by sediment is widely recognized as a significant driver of reservoir eutrophication, particularly in relation to nitrogen and phosphorus pollutants, the research conducted by Yi et al. has demonstrated the significant impact of MPs in sediments on nitrogen and phosphorus cycling processes^28^. And findings from the study conducted on Qinghai Lake also indicated a gradual release of diisooctyl phthalate into the aquatic environment through sediments^29^. However, in the case of rivers, sediments appeared to function as active agents in capturing MPs within the water, thereby significantly attenuating their release into the aquatic environment^30^.

Compared to water, sediments exhibited a greater abundance of MPs, ranging from 350 to 714 n/kg _(dw)_, with an average abundance of 490.83 n/kg _(dw)_ in main reservoir; and ranging from 299 to 1360 n/kg _(dw)_, with an average abundance of 586.00 n/kg _(dw)_ in river (Fig. 2). Consistent with previous studies, the sediment samples collected upstream of the reservoir exhibited a higher abundance of MPs compared to those downstream^12,31^, and the water velocity stands out as one of the primary factors contributing to this phenomenon. In contrast to river channels, the flow velocity of different water layers in reservoirs varies due to their larger water depth, with the bottom layer often exhibiting a lower velocity compared to the surface, and this differential flow velocity provides favorable conditions for the aggregation process of MPs into sediments^32,33^. Some researchers have also indicated the escalating tendency of MPs accumulation from water bodies to sediments^34,35^.

### Size of MPs

**Fig. 3:**
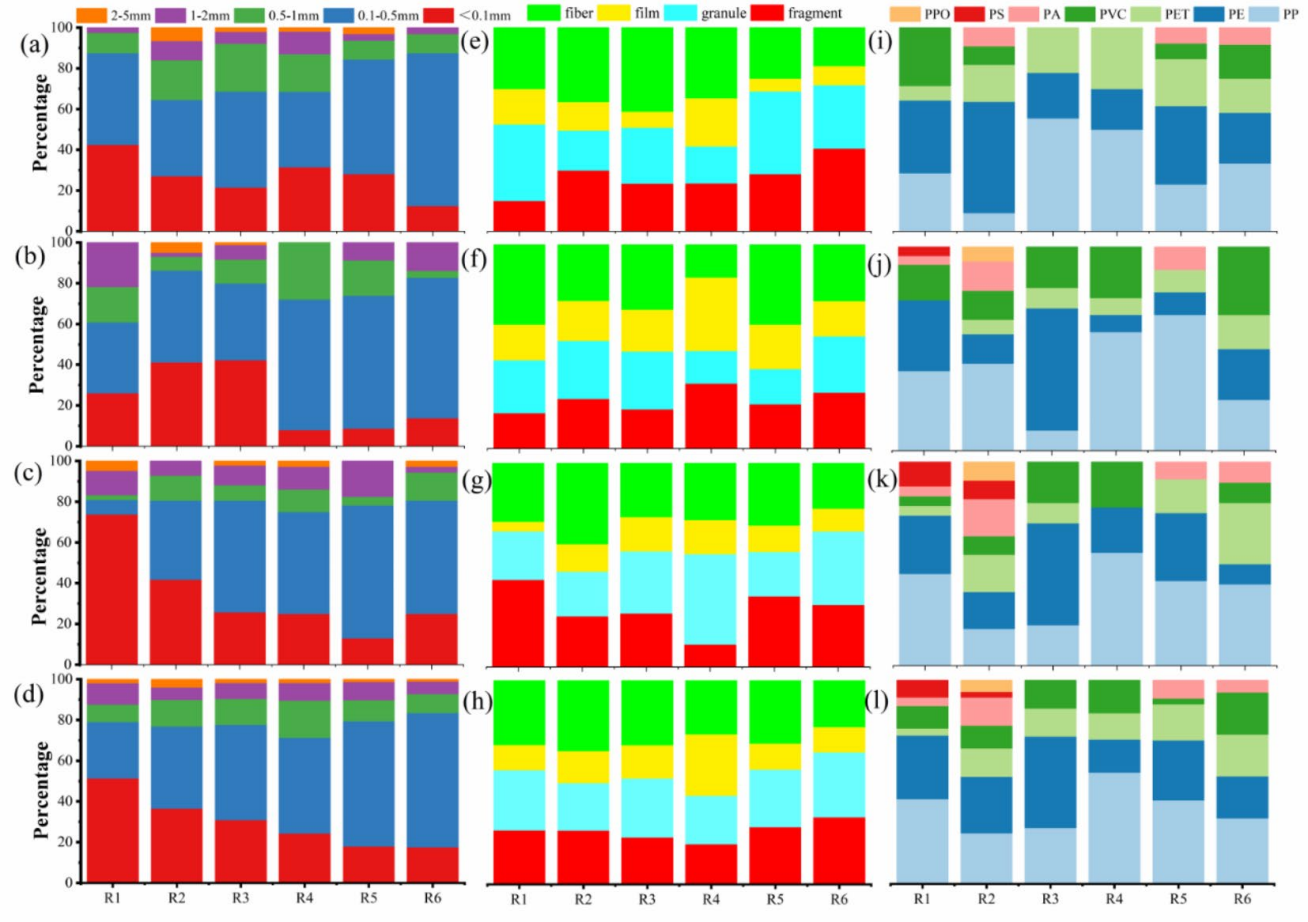
Percentage of MPs’s size, morphology and polymer type in reservoir (**a**, **e**, **i**: Surface water, **b**, **f**, **j**: Middle water, **c**, **g**, **k**: Bottom water, **d**, **h**, **l**: Totality).

The size distribution of MPs in the surface, middle, and bottom layers as well as the overall MP size distribution at each sampling point, is depicted in Fig. 3. It can be clearly seen that the water samples at different depths are dominated by the size of 0.1 to 0.5 mm, which accounted for 45.57%, 47.74%, and 44.21% in surface, middle, and bottom, respectively. The percentage of MPs of 0.1 to 0.5 mm size was 45.76% of all samples from the reservoir. The percentage of those smaller than 0.1 mm was 31.79%, and the smallest percentage of MPs was 2 to 5 mm, which was 2.36%. The results of MPs in river water samples are shown in Fig. S1b, the predominant sizes were 0.1 to 0.5 mm and < 0.1 mm, accounting for 58.46% and 17.95%, respectively, with over 85% of MPs measuring less than 1 mm.

In this study, it was observed that the proportion of MPs ranging from 0.1 to 0.5 mm gradually decreased from point R6 to point R1, while the proportion of less than 0.1 mm exhibited a gradual increase, potentially attributed to the hydrodynamic patterns within the reservoir. At the R1 to R3 point, where the reservoir and river converge, the enhanced flow velocity amplifies the shear force exerted by the current, leading to fragmentation of larger MPs into smaller particles^36^. Similar results were found in the studies of Jiayan Reservoir^31^, Liujiaxia Reservoir^15^, and Three Gorges Reservoir^14^.

The proportions of size in the reservoir sediments at each point are shown in Fig. S1a, the largest percentage of MPs was the size of 0.1 to 0.5 mm, and even 71.43% at the R5. The proportions of MPs in the reservoir sediments were as follows: 40.12% for ranging from 0.1 to 0.5 mm, 31.14% for ranging from 0.5 to 1 mm, and 20.36% for ranging from 1 to 2 mm. The predominant size fractions in river sediments were 0.1 to 0.5 mm (51.75%), < 0.1 mm (17.48%), and 0.5 to 1 mm (14.69%), respectively (Fig. S1c). In addition, it is noteworthy that no MPs smaller than 0.1 mm were found at point T1. The sediments were predominantly composed of MPs smaller than 1 mm, constituting over 70% of the total, which aligns with the findings observed in the water samples. The distance between T1 and R3 is approximately 4 km. The size distribution of MPs at T1 is illustrated in Fig. S1. Upon examining the raw data during the experiment, it was observed that MPs smaller than 0.1 mm accounted for nearly 20% in water samples at T1, but not in sediments. The sediment primarily consisted of MPs smaller than 1 mm, accounting for over 70% of the total composition, which is consistent with the findings observed in the water samples.

Interestingly, MPs smaller than 0.054 mm were detected in both water and sediment samples. During field sampling, a stainless steel screen with a diameter of 0.054 mm was utilized for filtration. Theoretically, MPs smaller than this size should not have been retained by the screen; however, previous studies have indicated that MPs, particularly those of microscopic dimensions, possess strong adsorption capabilities^37^. So we hypothesize that these MPs smaller than 0.054 mm might have adhered to certain particles and subsequently become trapped on the screen during sampling, only to be re-dispersed into the water during laboratory sample pretreatment. In a previous study, it was noted that MPs could migrate downwards through soil pores when their size was smaller than the pore diameter^38^. Additionally, smaller MPs exhibit stability in sediment, and their relative abundance increases with sediment depth^39^.

### Morphology of MPs

**Fig. 4:**
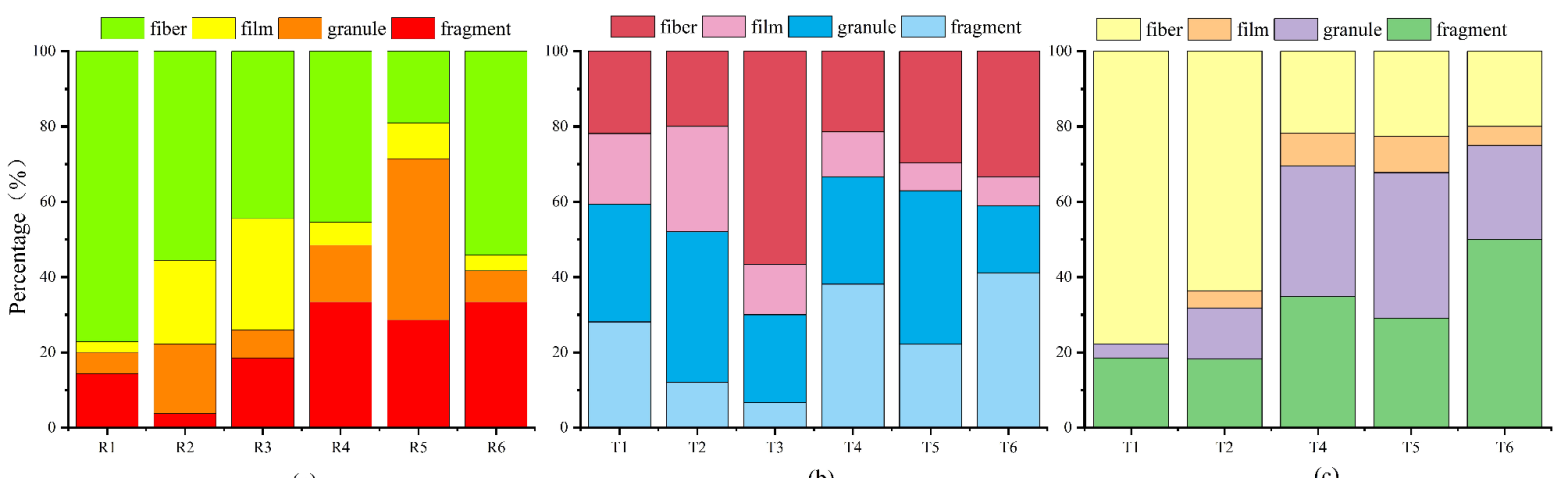
Percentage of morphology (**a**: reservoir sediment, **b**: river water, **c**: river sediment).

The MPs in all samples were classified into four categories: fragment, film, granule, and fiber, as shown in Figs. 3 and 4a, MPs in the reservoir and river water were predominantly in the form of fiber, accounting for 31.13% and 30.26% respectively. In the surface, middle, and bottom layers of the reservoir, fibrous MPs similarly represent the highest proportion, accounting for 33.12%, 29.66%, and 30.49% respectively. Granular MPs and fragmented MPs accounted for 27.06% and 25.88% of the total MPs in reservoir water samples, while granular MPs and fragmented MPs accounted for 29.23% and 26.67% of the total MPs in river water samples, respectively. Both reservoir and river water samples had the lowest percentage of film, accounting for 15.94% and 13.85% of the total MPs, respectively. Due to the presence of inhabited villages and aquaculture areas surrounding Yahekou Reservoir, anthropogenic activities constitute one of the primary sources of MPs within the reservoir. In previous study on the subject, it was noted that sewage plants treating wastewater are not able to trap and treat all of the MPs, thus resulting in a portion of the MPs being discharged with the sewage plant into the river^40^. Previous studies had indicated that the majority of fibrous MPs detected in freshwater ecosystems were likely derived from wastewater treatment plants^41^. Furthermore, it had been observed that certain reservoirs may also receive fibrous MPs originating from downstream wastewater treatment plant effluents, which were transported via rivers and subsequently accumulate within reservoirs. Additionally, investigations had demonstrated that fibrous MPs can be attributed to laundry wastewater as well^42^, Conversely, granular MPs primarily stem from household cleaning products and cosmetics, while fragmented MPs were predominantly a consequence of plastic product breakage such as disposable lunch boxes and plastic water bottles. Furthermore, particles generated from tire wear were introduced into the aquatic environment via stormwater runoff, which was also recognized as a significant source of particulate MPs^43^. The road infrastructure surrounding Yahekou Reservoir is well-developed, accommodating a high volume of vehicular traffic. Consequently, the abrasion of automobile tires generates MPs that can be transported into the reservoir through runoff. Additionally, tourist and fishing vessels employ tires as collision buffers, further contributing to the influx of granular MPs resulting from daily wear and tear. Notably, an extensive area of approximately 20,000 hectares worldwide was covered with mulch for short-term crop enhancement purposes; consequently, agricultural emissions represented a significant source of thin-film MPs^44,45^.

The morphology of MPs in reservoir and river sediment samples is depicted in Fig. 4b and c which also divided into four distinct categories. Notably, fibrous MPs dominate both the reservoir sediment (51.50%) and the river sediment (38.46%). Reservoir sediments were predominantly occupied by fibrous MPs (more than 40%), except for R5. The presence of aquaculture and fishing activities in the vicinity of the reservoir contributed to an increased abundance of fibrous MPs in both water and sediments. These findings align with previous research conducted on the Arras River and reservoir in northwestern Iran^46^. The R5 point, conversely, exhibited a prevalence of granules and fragments, constituting 42.86% and 28.57% respectively.

### Polymer types of MPs

Seven polymer types had been detected in all water samples including Polypropylene (PP), Polyethylene (PE), Polyethylene terephthalate (PET), Polyvinyl chloride (PVC), Polyamide (PA), Polystyrene (PS), and Polyphenylene oxiole (PPO) (Fig. 3i, j, k, and l). The surface layer of reservoir water samples was predominantly composed of PE, while the middle and bottom layers were dominated by PP, accounting for 33.33%, 40.59%, and 41.18% respectively. The higher proportions of MPs in Yahekou Reservoir were PE (38.89%), PP (29.41%), PET (10.13%), and PVC (12.09%), which is consistent with the findings in other reservoirs^14,15,31,47^. However, due to the diverse geographical environments in which reservoirs are situated, the predominant polymer types will vary accordingly^12^. The river water is also dominated by PE and PP, accounting for 50% and 26.25% respectively (Fig. S2). Wharves, villages, and fish farms are present in the primary reservoir area of Yanhekou Reservoir and along the upstream river. The local inhabitants inevitably utilize a substantial quantity of plastic bags, disposable lunch boxes, and plastic products, which serve as the principal sources of PP and PE. Research findings indicate that river transport accounts for 20% of MPs in the ocean, with fishing activities (such as discarded fishing nets, lines, and boats) contributing to half of this proportion. Consequently, it is imperative to recognize fishing-related practices as a significant source of MPs pollution in rivers and reservoirs^48^.

**Fig. 5:**
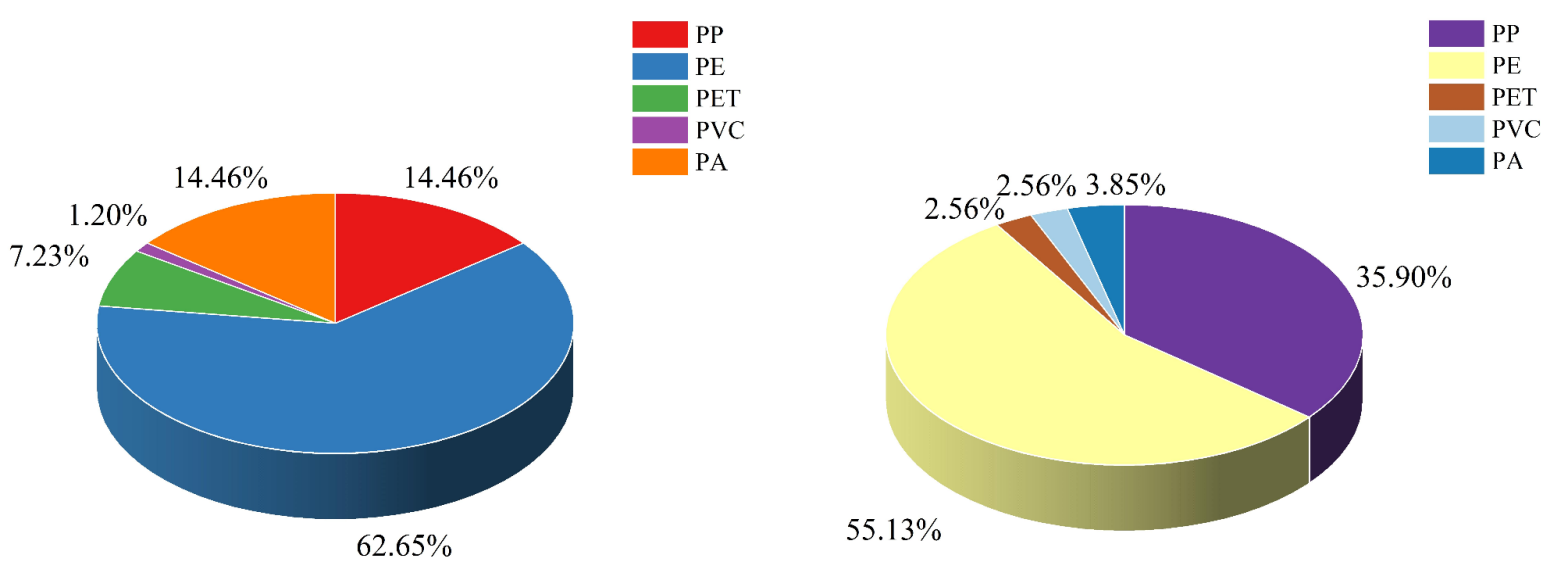
Percentage of polymer types in sediment (a: reservoir, b: river).

The percentage of polymer types in reservoir sediments is shown in Fig. 5a, where five polymer types like PE, PP, PET, PVC, and PA were detected in the sediments. Two polymers, PS and PPO, were absent compared to the water samples. The highest proportions of PE were observed in reservoir and river sediments, accounting for 62.65% and 55.13%, respectively, representing more than half of the total content (Fig. 5). Subsequently, PE constituted the next significant component. This finding is consistent with the results obtained from water samples, suggesting that the lower density of PP and PE compared to other polymer materials facilitates their transportation in sediments, potentially explaining their widespread occurrence in both water and sediments^32^. PP and PA account for the highest proportion in the sediment of Danjiangkou Reservoir^12^, PET and PP account for the highest proportion in the sediment of Liujiaxia Reservoir^15^, and PP and Cellulose account for the highest proportion in Jiayan Reservoir^31^. In these reservoirs, PP was found to have the highest proportion of MPs. This observation can be attributed to the affordability, versatile applications, and frequent daily usage of PP plastic, which is primarily utilized in the production of injection molded products, pipes, and boards. Notably, PS and PPO, two other types of polymers, were not detected in the sediment. However, despite their higher densities compared to PP and PE, they did not exhibit sedimentation. The migration process of MPs in aquatic environments is primarily influenced by both lateral and vertical movements, with the former being significantly affected by horizontal flow velocity. Vertical migration is determined not only by intrinsic factors such as density, size, and shape of MPs but also strongly influenced by extrinsic factors including turbulent conditions, sedimentation dynamics, and water quality^49^.

### Color of MPs

**Fig. 6:**
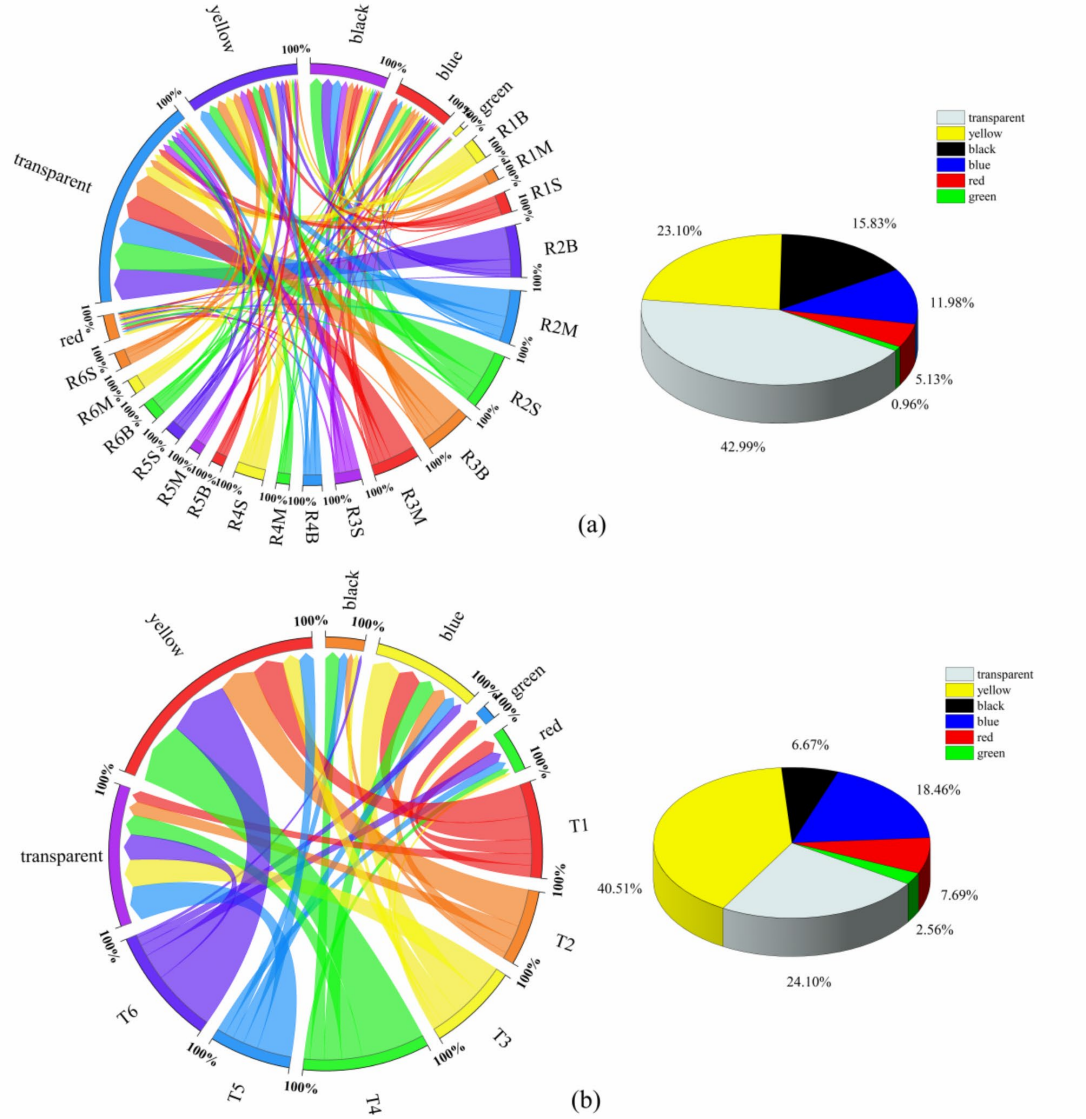
Percentage of MPs’ color in water (a: reservoir, b: river; S: Surface layer; M: Middle layer; B: Bottom layer).

When MPs were introduced into the aquatic environment, they were inadvertently or mistakenly ingested by aquatic organisms as a source of nutrition^50^. In a study conducted in a large natural lake, researchers found that black and blue MPs were the most frequently detected colors in fish samples. Furthermore, it was observed that MPs resembling food coloration may exhibit enhanced attractiveness, leading to preferential predation^51^. To further investigate the origin of MPs and determine whether the color composition of MPs in the reservoir influences their attractiveness to fish ingestion, thus entering the ecological chain, we conducted a comprehensive analysis on the color percentage within Yahekou Reservoir The color distribution of MPs at various points in Yahekou Reservoir is shown in Fig. 6a, and the most frequent color in the water samples was transparent, followed by yellow, which appeared in the ranges of 34.49 to 48.11% and 20.43 to 28.16%, respectively. The color percentage of MPs in the reservoir in proportional order was transparent (42.99%), yellow (23.10%), black (15.83), blue (11.98%), red (5.13%) and green (0.96%). In the river water samples, as shown in Fig. 6b, the largest proportion was yellow (40.51), followed by transparent (24.10%), which was slightly different from that in the reservoir water, where transparent and brown accounted for most of the samples in the Danjiangkou reservoir^12^.

The colors of MPs in reservoir sediments are shown in Fig. S3a. MPs were categorized into transparent, yellow, black, blue, red, and green within the sediments. Transparent (62.30%), black (17.40%) and blue (9.00%) accounted for more than 80% of the total. The same transparent MPs as those found in the water samples remained the most abundant, however, it is noteworthy that black and blue MPs also constituted a significant proportion of the sediments. Previous investigation had indicated that these particular colors were primarily derived from fishing nets and tires, which undergo long-term wear and tear resulting in the formation of fibrous MPs. These persistent fibers can persist within sediments for extended periods, thus being regarded as one of the prevalent environmental background pollutants^15^. In the sediments of Chitian Reservoir in Hainan, blue MPs were also more prevalent than yellow MPs^13^. The color composition of the river sediment was consistent with that of the water samples (Fig. S3b). The predominant hues were transparent and yellow, accounting for 58.04% and 16.08%, respectively. The proportion of green color was the lowest in both reservoir and river sediments, measuring 1.2% and 1.4%, respectively. A significant proportion of the reservoir and river exhibited heightened levels of transparency and yellow coloration, which can be attributed to the prevalent utilization of transparent plastic products in daily life. The significance of the percentage of yellow was also observed in this study, as transparent and white MPs undergo a yellowing process upon UV irradiation-induced aging^52,53^, and that the hydrogen peroxide solution selected during pretreatment also ages and discolors MPs^54^. Additionally, MPs undergo color alteration following weathering, such as transitioning from a pristine white hue to shades of yellow or black^55^. Therefore, this analysis suggests that the predominance of the yellow can be attributed to this factor. Other colors of MPs may primarily originate from colored items such as plastic bags and clothing.

### Risk assessment of MPs

**Fig. 7:**
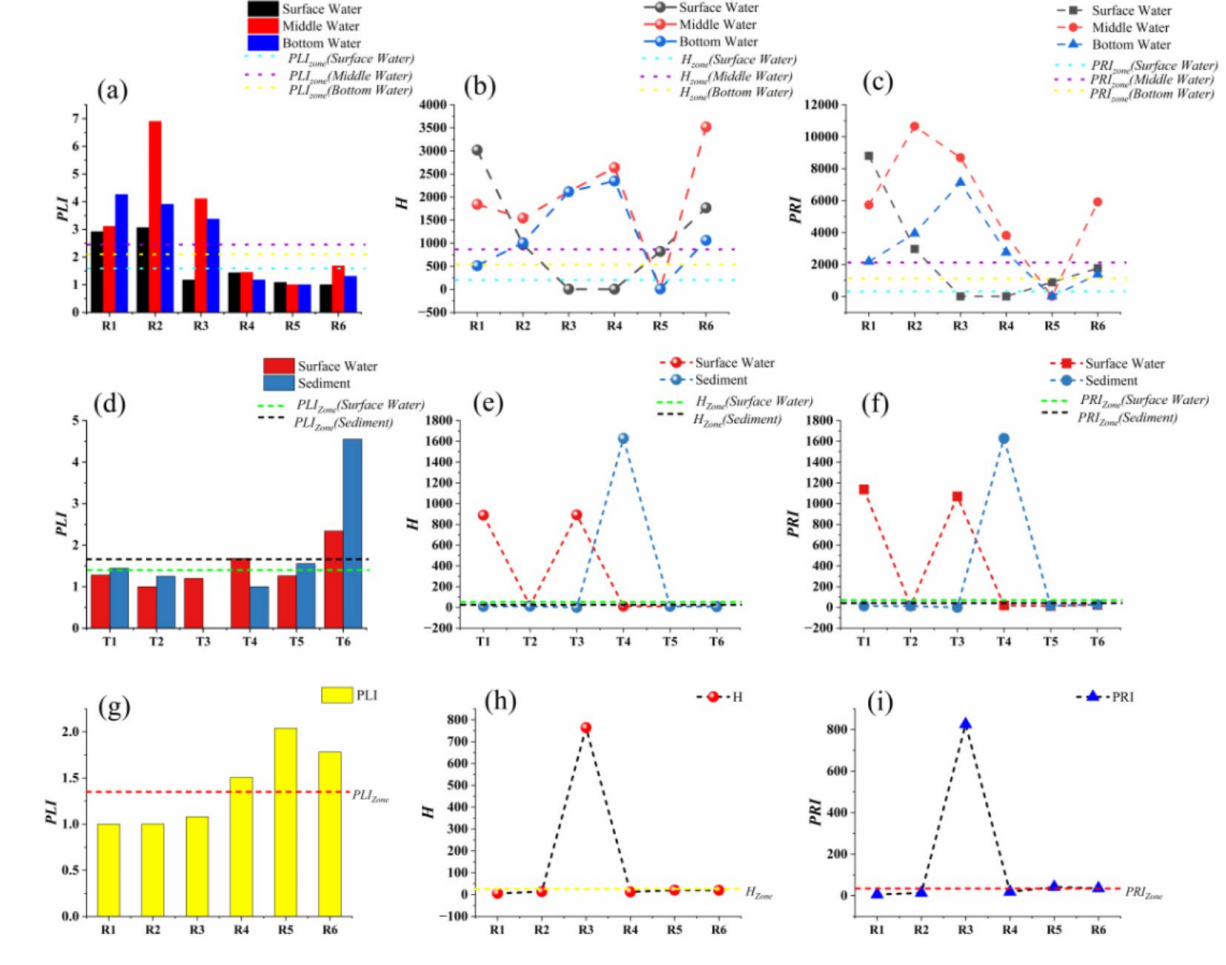
Risk assessment of MPs in Yahekou Reservoir and river (**a**, **b**, **c**: PLI, H, and PRI of surface, middle, and bottom water in reservoir; **d**, **e**, **f**: PLI, H, and PRI of water and sediment in river; **g**, **h**, **i**: PLI, H, and PRI of sediment in reservoir; PLI: Pollution Load Index, H: Hazard Index, PRI: Pollution Risk Index).

The pollution degree of MPs was assessed using PLI, H and PRI to evaluate the risk in surface, middle, bottom, and sediments of main reservoir as well as in the water and sediment of the upstream river. The sampling points exhibited significant differences, as depicted in Fig. 7. The calculated results indicate that the PLI values of the surface, middle, and bottom water in R4 to R6 were all below 2, classifying them as exhibiting light pollution. With the exception of the surface water in R3, the PLI values for the remaining three points exceed 2, indicating severe pollution. Notably, the PLI value for samples from R2 was significantly higher at 6.9 compared to other samples. Furthermore, PLI_Zone_ values were observed across different water layers: 1.59 for surface water, 2.45 for middle water, and 2.1 for bottom water. In general, MPs pollution was more prevalent in middle and bottom water layers compared to surface water. Furthermore, a positive correlation can be observed between the change in PLI value and abundance, indicating that areas with higher PLI values are associated with more intense human activities near the sampling points. Compared with Chitian Reservoir and Liujiaxia Reservoir, the level of MPs contamination in Yakhekou Reservoir was higher. The PLI value of the river reached its peak at T6, measuring 2.34, while the PLI_Zone_ value was 1.4 (Fig. 7d). When comparing the PLI values of river and reservoir water, it could be observed that overall, the PLI value of river water is slightly lower than that of reservoir water. Considering the variations in water depth and flow velocity between river and reservoir, with typically higher flow velocities in river, a negative correlation between flow velocity and MPs abundance had been confirmed in the previous study^27^. The pollution levels in different water depths within reservoirs indicated that MPs exhibited a predominant distribution in the middle and bottom water. The PLI_Zone_ value of the sediments was 1.35, which is comparatively lower than that observed in bottom water. However, the PLI_Zone_ in river sediment (1.66) surpasses that observed in river water samples, indicating a comparatively accelerated settling of MPs in river as opposed to reservoir.

The PLI solely assesses pollution levels based on the abundance of MPs. However, it is imperative to incorporate H value in order to further investigate the toxicological implications of MPs as chemical polymers^26^. The specific hazard indices for each sampling point are shown in Fig. 7b, e, and h. The values of H for surface water, middle water, bottom water and sediment in the reservoir ranged from 3.89 to 3019.1, 7.56 to 3520.67, 8.67 to 2347.67, and 5.6 to 764 with H_Zone_ of 200.2, 866.47, 535.77 and 25.55, respectively. The value of H for middle and bottom water were significantly higher, with the middle water exhibiting the highest value of R6 at 3520.67, while the bottom water recorded R4 at 2347.67. Both values fall within the risk class IV category. The values of H for river water and sediments, as well as the range of 10 to 891.17 and 5.88 to 1628.15, respectively, were comparatively low in comparison to the reservoirs. Additionally, the values of H_Zone_ for river water and sediments were 49.93 and 25.09, respectively. The poor agreement between PLI and H can be mainly attributed to the varying hazard scores of different polymers in the ecosystem. For instance, while PP has a hazard score of only 1, PVC exhibits a significantly higher hazard score of 10,551. Consequently, the presence of PVC at collection sites with its substantially elevated hazard index distinguishes it from other collection sites by thousands-fold difference. A previous report has suggested that the high toxicity score of polymers greatly influences the H value^56^.

The PRI proposed by Hakanson in 1980^14^, which combines PLI and H for ecological risk assessment. The results of PRI at each point within the main reservoir area are presented in Fig. 7c and i. It is evident that PRI exhibits similar trends to H, with higher values of sediment observed at the R3 point. Specifically, PRI_Zone_ values were determined as 318.64, 2120.31, 1125.34, and 34.39 for surface water, middle water, bottom water, and sediment respectively. Notably, all three water layers exhibited high pollution levels according to hazard risk categorization while the sediment showed slight contamination. The PRI_Zone_ values for river water and sediments in the index assessment were 70.02 and 41.76, respectively, both indicating a moderate level of risk. The underlying reason for this phenomenon can be attributed to the inherent association between PRI and H during computation, whereby sites with polymers of high hazard scores also exhibit increased PRI values. For instance, in the middle water at R6, the PLI_6_ was 1.68, H_6_ reaches 3520.67, and PRI_6_ soared as high as 5918.8; moreover, PVC with high hazard scores accounts for 33.33% of the composition at this location, rendering it susceptible to hazardous contamination. At T6 point, PLI_6_ was 4.55 while H_6_ was 5.88; consequently, PRI_6_ amounted to a modest value of 26.8 indicating a slight risk of contamination at this site specifically. The PLI_Zone_, H_Zone_ and PRI_Zone_ for reservoir water samples were 2.05, 534.08 and 1188.1, respectively; and for river water samples were 1.4, 49.93 and 70.02, respectively. The PLI_Zone_, H_Zone_ and PRI_Zone_ for reservoir sediment samples were 1.35, 25.55 and 34.39, respectively; and for river sediment samples were 1.66, 25.09 and 41.76, respectively. In conclusion, both PLI and H contributed significantly to the determination of the pollution risk index; however, it is imperative to acknowledge that the polymer’s hazard score plays a pivotal role.

Yakhekou Reservoir serves as a crucial drinking water source in Nanyang City, ensuring the safety of drinking water for the local population’s daily lives. Therefore, it is imperative to address the significant MPs pollution levels, minimize the utilization of plastics with high hazard scores, and enhance awareness regarding the impact and risks posed by MPs on water safety and ecological environment. Furthermore, there is a pressing need to explore novel methodologies for risk assessment of MPs. Currently; the absence of standardized approaches and models hinders accurate evaluation of ecological risks associated with MPs in aquatic environments. Solely considering the abundance of MPs and chemical hazards may underestimate their true ecological implications.

###  Relationship between MPs abundance and water quality

**Fig. 8:**
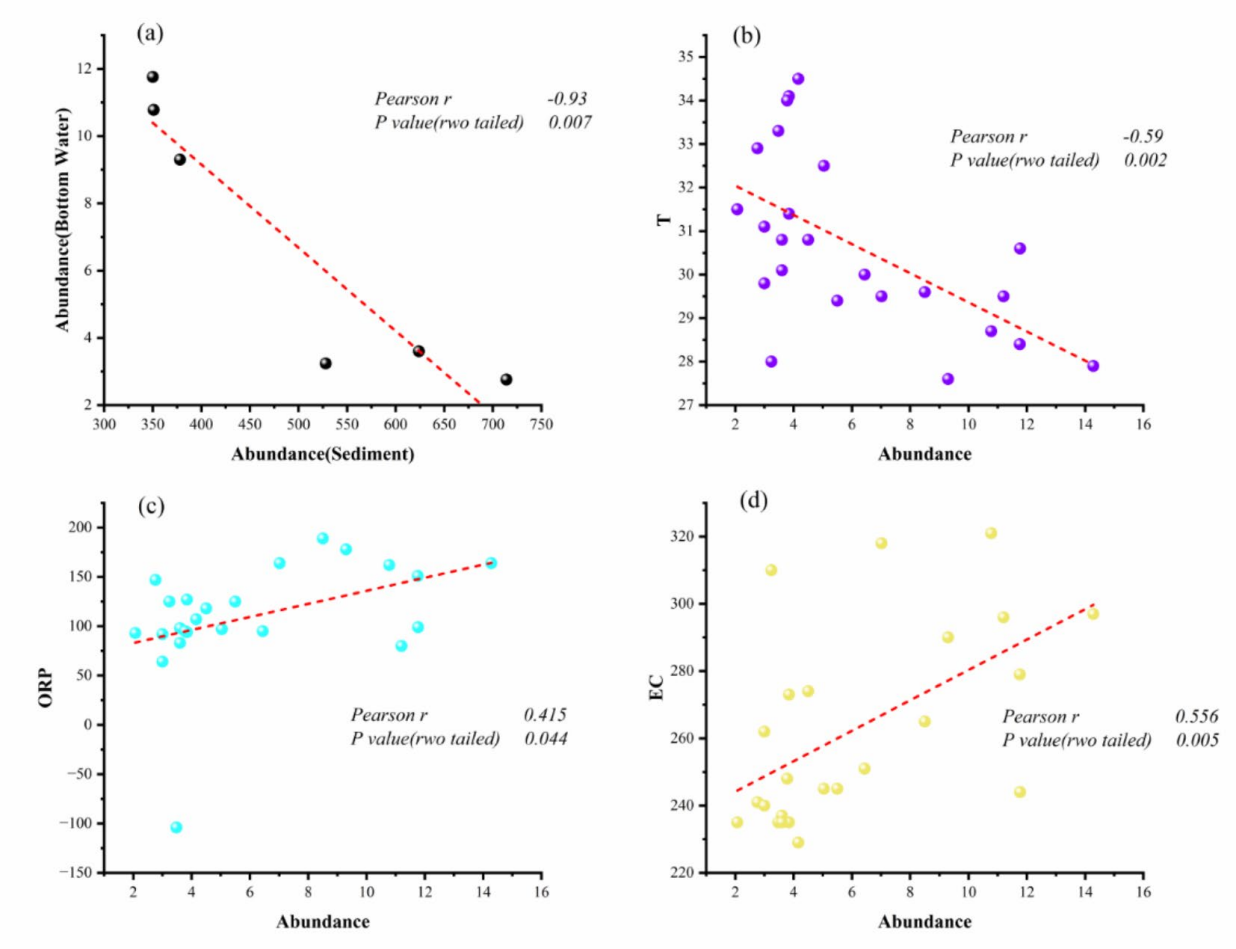
Correlation fitting between MPs abundance in Yahekou Reservoir and water quality parameters (**a**: Abundance of MPs between bottom water and sediment in reservoir, **b**: The relationship between MPs and T, **c**: The relationship between MPs and ORP, **d**: The relationship between MPs and EC; T: temperature, ORP: oxidation reduction potential, EC: electrical conductivity).

Currently, the pretreatment procedures for water or sediment samples containing MPs are intricate, and the identification and analysis of MPs typically entail a substantial amount of time. Therefore, by examining the correlation between MPs abundance and water quality indices, we can gain an initial understanding of MPs content in samples to facilitate subsequent investigations^20^. Surface water abundance in Yahekou Reservoir was correlated with sediment abundance and found not to be significantly correlated (*P* > 0.05). Nguyen had the same results in their study on the Ma River in Vietnam, the discrepancy in transport mechanisms between the two media may account for this outcome, while it is also crucial to consider that variations in sampling techniques employed for samples could potentially contribute to disparities in the data^57^. Interestingly, we observed a significant negative correlation between the abundance of MPs in the bottom water and sediment, after correlating it with the abundance of middle and bottom water in the reservoir (*P < 0.05*, *r =-0.93*) (Fig. 8a). Considering the variation in current velocity at different depths, it is important to acknowledge that the velocity of water flow also plays a significant role in influencing the abundance of MPs. The current study still has limited reports on MPs in different water layers in lakes and reservoirs with large water depths. Instead, researchers conducted a correlation analysis between the abundance of MPs in surface water and sediments, yielding contrasting findings compared to those obtained in this study^58^. The negative correlation observed between MPs in bottom water and sediments may be attributed to a significant presence of small-sized MPs suspended in middle and bottom water, while larger particles are more prone to sinking into sediments. Figure 3c and S1c demonstrate that there is a higher proportion of large-size MPs found in sediments compared to water samples. Another potential explanation could be sample limitations resulting from single sampling carried out within this study. therefore, continuous sampling is necessary to expand the sample size for a more accurate representation of correlations.

To further investigate the relationship between other water quality parameters and MPs abundance, we conducted a Pearson’s correlation analysis on the water quality parameters. The water quality parameters included Oxidation-Reduction Potential (ORP), Electrical Conductivity (EC), pH, Dissolved Oxygen (DO), Turbidity, and Temperature (T). The results revealed significant correlations between MPs abundance in surface water and ORP, EC, and T (*P < 0.05*,*r = 0.42*,*r = 0.56*, and *r* = -0.59, respectively) (Fig. 8b, c, and d). The remaining water quality parameters did not show correlation with MPs abundance (Fig. S4). Physicochemical properties such as turbidity, electrical conductivity, total solids and biological oxygen demand (BOD) were found to have a strong influence on the distribution of MPs in a study of urban canals in Thailand^59^. The study conducted by Li et al. in various lakes situated in the middle and lower regions of the Yangtze River, including Dongting Lake, revealed a significant and positive correlation between the sediment MPs abundance and the mass concentration of total nitrogen (TN) present in lake water^25^. The study on MPs in the Brentas River, East Java, Indonesia revealed that temperature, BOD, total suspended solids (TSS), and turbidity were identified as the most influential physicochemical parameters impacting the abundance of MPs^60^. In this study, we found a negative correlation between temperature and the abundance of MPs. This can be attributed to various factors such as hydrodynamic conditions specific to comprehensive reservoirs, surrounding environmental influences on the reservoir, and the predominant polymer types present in the water column. Specifically, smaller-sized MPs tend to agglomerate and deposit due to their increased likelihood of interaction with each other despite having a larger specific surface area. Conversely, larger-sized MPs primarily deposit under gravitational forces leading to an observed negative correlation.

## Conclusion

In this study, we conducted field sampling of water and sediment samples from 12 designated sampling points in the Yanhekou Reservoir and river, enabling the identification of MPs content within them. The abundance of MPs in the reservoir water samples ranged from 2.07 n/L to 14.28 n/L, and the average abundance of MPs in surface, middle, and bottom waters were 6.83 n/L, 6.30 n/L, and 6.91 n/L, respectively, and the abundance of MPs in sediment samples ranged from 350 to 714 n/kg, significant differences between points were found by one-way ANOVA. Both water and sediment samples in the main reservoir were dominated by < 0.5 mm size transparent fibers. A total of seven polymer types of MPs were identified in the reservoir, with PP and PE being the predominant types, constituting over 50% of both water and sediment samples. The high prevalence of PP and PE in the samples can be primarily attributed to daily human activities. The abundance of MPs in river water samples ranged from 3 to 7.02 n/L, with an average abundance of 4.38 n/L. Meanwhile, the abundance of river sediment samples varied from 299 to 1360 n/kg, with an average abundance of 586 n/kg. Fibrous MPs smaller than 0.5 mm dominated both river water and sediment samples, with yellow being the predominant color in river water and transparent being the dominant color in river sediments. A total of five polymer types were identified in the rivers, primarily PE and PP. Furthermore, a preliminary ecological risk assessment was conducted on the surface, middle, bottom water, and sediments of Yahekou Reservoir by evaluating PLI, H, and PRI. The PLI_Zone_, H_Zone_ and PRI_Zone_ for reservoir water samples were 2.05, 534.08 and 1188.1, respectively; and for river water samples were 1.4, 49.93 and 70.02, respectively. The PLI_Zone_, H_Zone_ and PRI_Zone_ for reservoir sediment samples were 1.35, 25.55 and 34.39, respectively; and for river sediment samples were 1.66, 25.09 and 41.76, respectively. The findings revealed that the surface water exhibited a moderate level of risk while the sediments posed a low level of risk. However, both middle and bottom water demonstrated hazardous levels of risk due to higher concentrations of polymers with significant toxicity indices in these reservoir. The correlation analysis results revealed significant associations between MPs abundance and water quality parameters, including ORP, T, and EC. Moreover, a significant negative correlation was observed between MPs abundance and T. The present study investigates the current status and ecological risk level of MPs contamination in the Yakhekou Reservoir, providing valuable data on MPs contamination in source reservoirs within the Yangtze River Basin. These findings offer crucial support for future management and protection strategies aimed at safeguarding the Yakhekou Reservoir.

## Limitations

There are still certain limitations to this study. Firstly, the short timeline of this study is attributed to the management policy of the study site. Additionally, in the pretreatment stage, due to concerns regarding environmental harm caused by the experimental drug, we opted for saturated sodium chloride solution as the flotation agent, which may result in some loss of flotation quantity. Moreover, utilization of hydrogen peroxide solution will inevitably impact the MPs sample itself, leading to potential quality degradation and color alteration. We are actively addressing these issues and our organization is currently engaged in a collaborative agreement with the reservoir management department to conduct an extensive investigation into spatial and temporal variations of MPs in Yahekou Reservoir. Furthermore, we aim to establish a standardized set of methods for pretreating MPs samples from source water through collaboration with other researchers so that results from different sites can be compared within a consistent framework.

## Electronic supplementary material

Below is the link to the electronic supplementary material.

Supplementary Material 1

## Data Availability

The corresponding author affirms that the data will be made available upon request.
