# Supplementary material for: Distribution and risk assessment of microplastics in a source water reservoir, Central China
Source: Sci Rep. 2025 Jan 2;15:468. doi: 10.1038/s41598-024-84894-z (PMC11695635; doi:10.1038/s41598-024-84894-z)
Supplement: Supplementary file 1 — Supplementary Material 1 [file 41598_2024_84894_MOESM1_ESM.docx]

Supplementary Materials

Distribution and risk assessment of microplastics in a source water reservoir, Central China

Minghui Shen, Yang Li*, Liwen Qin, Xudong Chen, Tianyu Ao, Xishu Liang, Kaibo Jin, Yanyan Dou, Juexiu Li, Xuejun Duan

School of Smarts Energy and Environment, Zhongyuan University of Technology, Zhengzhou, 450007, China

Correspondence: liyang@zut.edu.cn

**Table of Contents**

**Table S1**. Latitude and longitude coordinates of sampling points

**Table S2**. Pollution Load Index Pollution Levels and Risk Categories

**Table S3**. Toxicity scores of common polymers

**Table S4.** Details of water quality parameters at each point

**Table S5.** Proportion of colors at different depths at various points of the reservoir

**Figure S1**. Percentage of MPs’ size in river water, sediment and reservoir sediment (a: reservoir sediment，b: river water，c: river sediment)

**Figure S2**. Percentage of polymer types in river sediment

**Figure S3**. Percentage of MPs’ color in sediment (a: reservoir, b: river)

**Figure S4**. Correlation between MPs abundance and water quality parameters

**Figure S5**. Electron micrograph of MPs (fragment: a, d, g; granule: b, e; fiber: c, f; film: h, i;)

**Figure S6**. Raman spectra of various polymers

**Table S1**. Latitude and longitude coordinates of sampling points

| Sampling site | Longitude | Latitude |
| --- | --- | --- |
| R1 | 112°36′12″ | 33°17′57″ |
| R2 | 112°36′32″ | 33°18′16″ |
| R3 | 112°36′55″ | 33°18′28″ |
| R4 | 112°36′06″ | 33°18′36″ |
| R5 | 112°35′11″ | 33°19′15″ |
| R6 | 112°34′03″ | 33°19′56″ |
| T1 | 112°38′02″ | 33°19′26″ |
| T2 | 112°33′44″ | 33°20′35″ |
| T3 | 112°32′24″ | 33°20′22″ |
| T4 | 112°29′57″ | 33°21′47″ |
| T5 | 112°30′06″ | 33°22′18″ |
| T6 | 112°38′15″ | 33°16′48″ |

**Table S2.** Pollution Load Index Pollution Levels and Risk Categories

| PLI |  | PRI | Risk category | H | Risk category |
| --- | --- | --- | --- | --- | --- |
| >1 | Polluted | ＜150 | Minor | ＜10 | Ⅰ |
|  |  | 150-300 | Medium | 10-100 | Ⅱ |
|  |  | 300-600 | Considerable | 100-1000 | Ⅲ |
|  |  | 600-1200 | High | 1001-10000 | Ⅳ |
|  |  | ＞1200 | Danger | ＞10000 | Ⅴ |

**Table S3.** Toxicity scores of common polymers

| Polymers | Toxicity score |
| --- | --- |
| PP | 1 |
| PE | 11 |
| PET | 4 |
| PVC | 10551 |
| PA | 47 |
| PPO | 400 |
| PS | 30 |

**Table S4.** Details of water quality parameters at each point

|  | ORP | pH | EC | DO | T | NTU |
| --- | --- | --- | --- | --- | --- | --- |
| R1S | 80 | 8.9 | 296 | 7.5 | 29.5 | 2.92 |
| R1M | 95 | 8.5 | 251 | 6.2 | 30 | 2.02 |
| R1B | 151 | 8.3 | 279 | 4.6 | 28.4 | 2.2 |
| R2S | 99 | 8.9 | 244 | 7.3 | 30.6 | 3.11 |
| R2M | 164 | 8.2 | 297 | 4.4 | 27.9 | 4.15 |
| R2B | 162 | 8.4 | 321 | 4.7 | 28.7 | 1.83 |
| R3S | 118 | 8.6 | 274 | 7.1 | 30.8 | 2.52 |
| R3M | 189 | 8.2 | 265 | 3.9 | 29.6 | 2.01 |
| R3B | 178 | 8.3 | 290 | 4.2 | 27.6 | 1.96 |
| R4S | 125 | 9 | 245 | 7.4 | 29.4 | 3.33 |
| R4M | 64 | 8.2 | 262 | 4.5 | 29.8 | 3.4 |
| R4B | 125 | 8.1 | 310 | 4.5 | 28 | 2.2 |
| R5S | 107 | 8.9 | 229 | 7.1 | 34.5 | 2.94 |
| R5M | 93 | 8.8 | 235 | 6 | 31.5 | 3.32 |
| R5B | 147 | 8.1 | 241 | 3.9 | 32.9 | 3.25 |
| R6S | 94 | 9 | 235 | 3.1 | 34.1 | 6.26 |
| R6M | -104 | 9 | 235 | 7.1 | 33.3 | 3 |
| R6B | 83 | 8.6 | 237 | 3.2 | 30.1 | 1.75 |
| T1 | 127 | 8.8 | 273 | 6.7 | 31.4 | 4.38 |
| T2 | 92 | 9 | 240 | 8.8 | 31.1 | 2.87 |
| T3 | 98 | 8.9 | 235 | 8.7 | 30.8 | 3.18 |
| T4 | 97 | 8.8 | 245 | 7.8 | 32.5 | 3.33 |
| T5 | 95 | 8.6 | 248 | 6.3 | 34 | 2.75 |

(R: Reservoir; T: River; S: Surface layer; M: Middle layer; B: Bottom layer)

**Table S5.** Proportion of colors at different depths at various points of the reservoir

| - | transparent | yellow | black | blue | red | green |
| --- | --- | --- | --- | --- | --- | --- |
| 1S | 33% | 30% | 23% | 13% | 3% | 0% |
| 1M | 61% | 13% | 9% | 9% | 9% | 0% |
| 1B | 52% | 17% | 19% | 5% | 5% | 2% |
| 2S | 51% | 18% | 23% | 5% | 3% | 0% |
| 2M | 52% | 22% | 19% | 2% | 5% | 1% |
| 2B | 64% | 8% | 21% | 6% | 1% | 0% |
| 3S | 26% | 28% | 26% | 8% | 8% | 4% |
| 3M | 54% | 15% | 10% | 15% | 6% | 1% |
| 3B | 51% | 21% | 14% | 10% | 2% | 2% |
| 4S | 25% | 33% | 16% | 13% | 11% | 2% |
| 4M | 52% | 20% | 20% | 4% | 4% | 0% |
| 4B | 22% | 33% | 11% | 31% | 3% | 0% |
| 5S | 31% | 34% | 3% | 22% | 9% | 0% |
| 5M | 43% | 22% | 13% | 22% | 0% | 0% |
| 5B | 35% | 35% | 13% | 17% | 0% | 0% |
| 6S | 13% | 47% | 6% | 25% | 9% | 0% |
| 6M | 7% | 41% | 0% | 38% | 14% | 0% |
| 6B | 14% | 36% | 6% | 28% | 14% | 3% |

(S: Surface layer; M: Middle layer; B: Bottom layer)





**Figure S1.** Percentage of MPs’ sizes in river water, sediment and reservoir sediment (a: reservoir sediment，b: river water，c: river sediment)





**Figure S2**. Percentage of polymer types in river sediment





**Figure S3**. Percentage of MP’ color in sediment (a: reservoir, b: river)


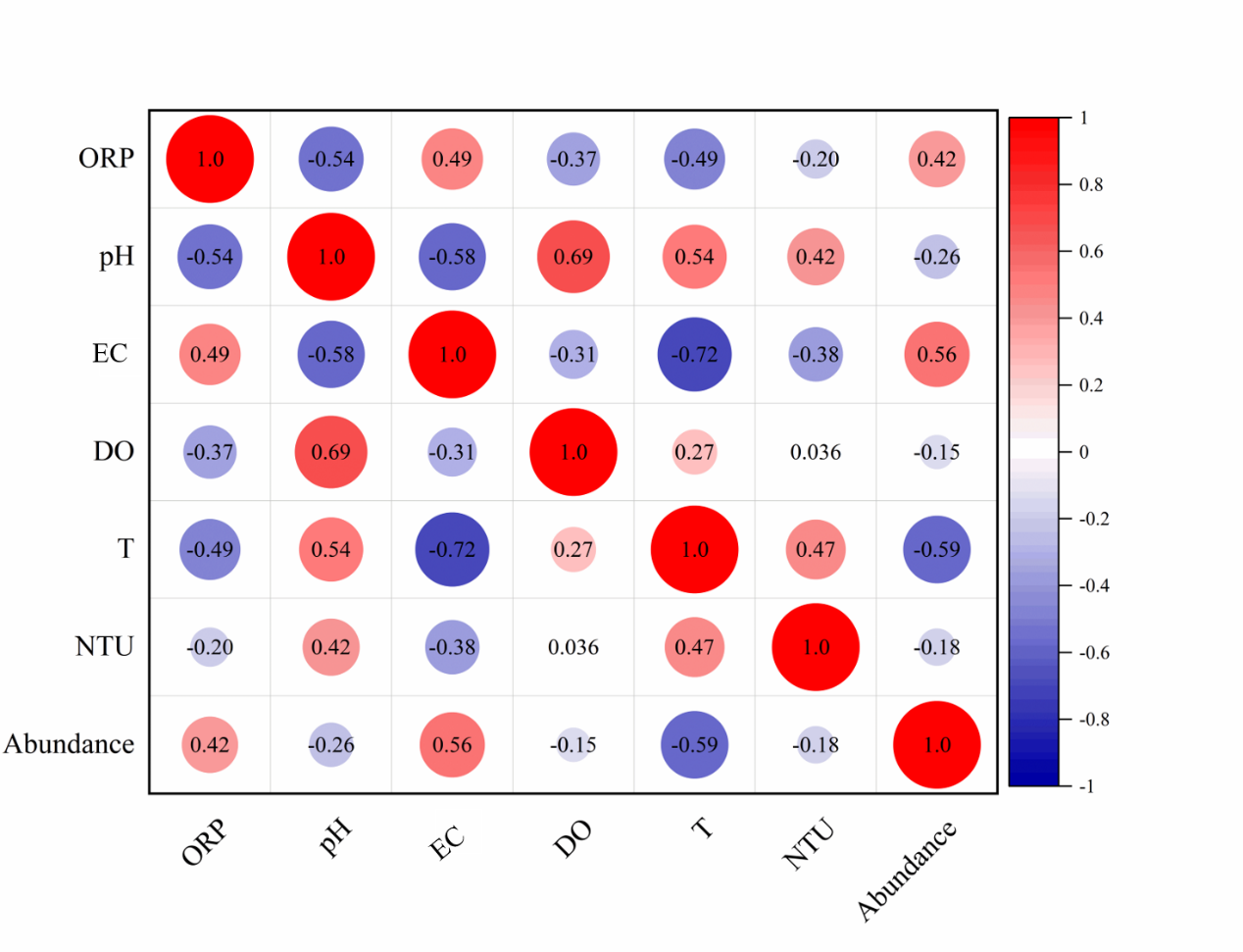


**Figure S4**. Correlation between MPs abundance and water quality parameters


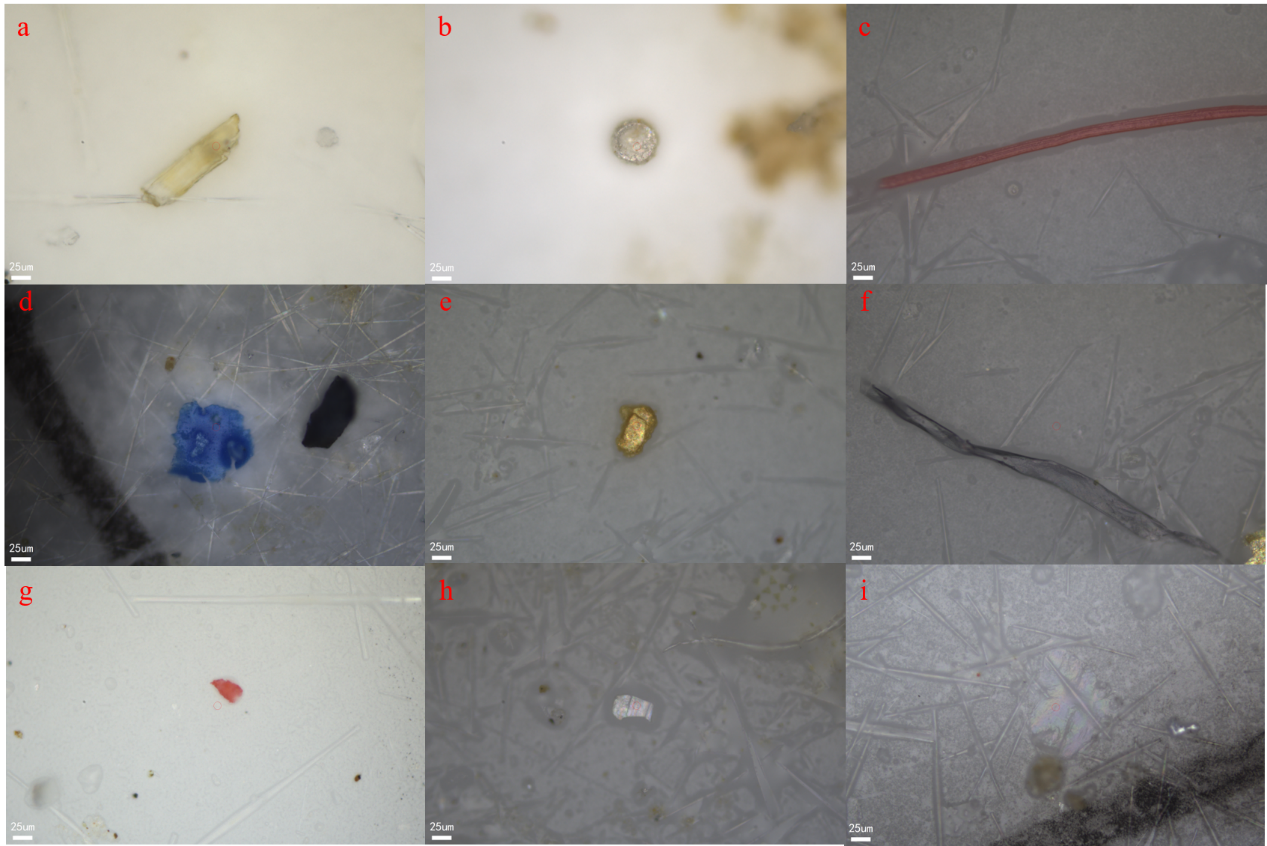


**Figure S5**. Electron micrograph of MPs (fragment: a, d, g; granules: b, e; fiber: c, f; film: h, i)





**Figure S6**. Raman spectra of various polymer.
